# Lemierre’s syndrome with septic pulmonary emboli

**DOI:** 10.11604/pamj.2024.47.188.43090

**Published:** 2024-04-16

**Authors:** Ashwin Karnan

**Affiliations:** 1 Department of Respiratory Medicine, Datta Meghe Institute of Higher Education and Research, Sawangi (Meghe), Wardha, Maharashtra, India

**Keywords:** Pharyngitis, jugular vein, thrombosis, embolism

## Image in medicine

A 32-year-old male presented to the emergency department with complaints of breathing difficulty, chest pain, and fever for the past 2 days. The patient gave a history of pharyngitis 3 weeks back, for which he was treated with oral antibiotics. Chest X-ray of the patient showed bilateral nodular opacities, with suspicion of cannonball metastases or pulmonary embolism. Contrast-enhanced computed tomography (CT) showed an intraluminal filling defect of the right internal jugular vein with bilateral nodular opacities in the lung with positive feeding vessel signs suggestive of septic pulmonary emboli. Blood culture showed methicillin-resistant *Staphylococcus aureus* growth. The patient was treated with appropriate intravenous antibiotics for 21 days, anticoagulants, and oxygen support. The patient improved symptomatically and is currently on follow-up. Lemierre’s syndrome is a rare disease with high mortality, characterized by thrombosis of the internal jugular vein and bacteremia following a recent oropharyngeal infection. The causative organism is thought to spread directly from the peritonsillar space to the jugular vein, with subsequent septic thromboembolism. Contrast-enhanced computed tomography is the gold standard investigation of choice. Treatment usually involves intravenous antibiotics.

**Figure 1:**
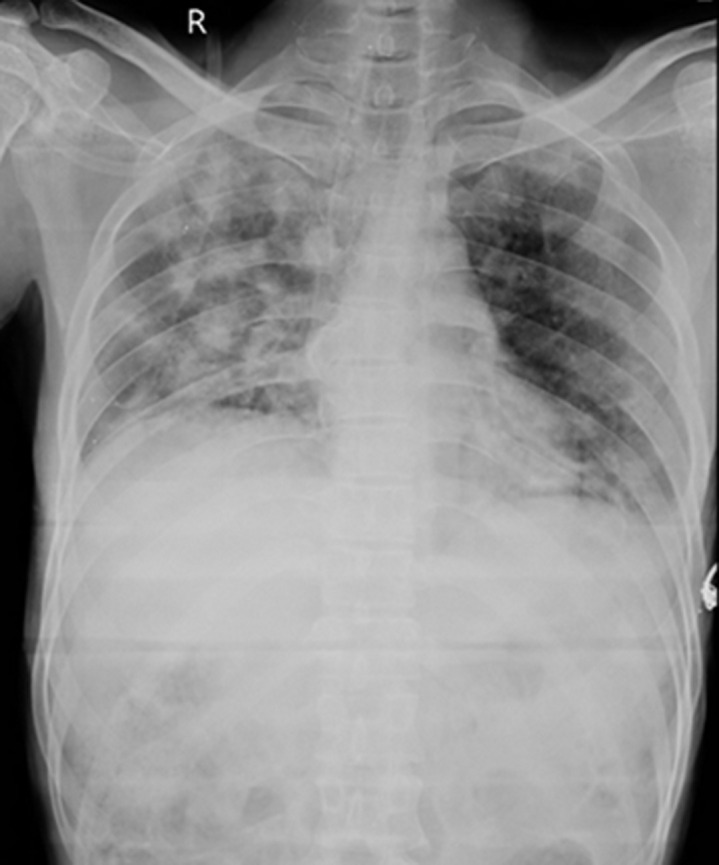
chest X-ray of the patient showing bilateral asymmetrical nonhomogeneous nodular opacities

